# Cross Sectional and Longitudinal Associations between Cardiovascular Risk Factors and Age Related Macular Degeneration in the EPIC-Norfolk Eye Study

**DOI:** 10.1371/journal.pone.0132565

**Published:** 2015-07-15

**Authors:** Jennifer L. Y. Yip, Anthony P. Khawaja, Michelle P. Y. Chan, David C. Broadway, Tunde Peto, Adnan Tufail, Robert Luben, Shabina Hayat, Amit Bhaniani, Nicholas J. Wareham, Kay-Tee Khaw, Paul J. Foster

**Affiliations:** 1 Department of Public Health & Primary Care, University of Cambridge, Cambridge, United Kingdom; 2 NIHR Biomedical Research Centre at Moorfields Eye Hospital and UCL Institute of Ophthalmology, London, United Kingdom; 3 Department of Ophthalmology, Norfolk and Norwich University Hospital, Norwich, United Kingdom; 4 MRC Epidemiology Unit, University of Cambridge School of Clinical Medicine, Cambridge, United Kingdom; University of Florida, UNITED STATES

## Abstract

**Purpose:**

To examine the cross sectional and longitudinal relationship between cardiovascular risk factors and age-related macular degeneration (AMD) in a large British cohort study.

**Methods:**

The EPIC Norfolk Eye study is nested in a larger prospective cohort study. Data on cardiovascular risk factors were collected at baseline (1993-1997) and follow up (2006-2011) via clinical examination, validated lifestyle questionnaires and serum blood samples. AMD was ascertained using standardised grading of fundus photographs at the follow up. Logistic regression was used to examine associations between baseline and follow up risk factors with AMD.

**Results:**

5,344 pairs (62.0% of total 8623) of fundus photographs were of sufficient quality for grading of AMD in participants with mean age of 67.4 years old (range 44-91) at diagnosis. There were 28 cases of late AMD (0.5%, 95% confidence interval (CI)=0.3-0.8%) and 645 cases of early AMD (12.1%, 95%CI=11.2-13.0.%). In multivariable analysis, older people with higher levels of baseline high density lipoprotein- cholesterol (HDL-C ) and C-reactive protein (CRP) were more likely to have any signs of AMD, after adjusting for sex, education, smoking, and systolic blood pressure. In cross sectional analysis, only older age and higher HDL were significantly associated with AMD.

**Conclusions:**

We have found that older age and higher levels of CRP and HDL-C were associated with increased odds of AMD in this population in the longitudinal analysis, but older age and HDL-C, not CRP was significantly associated with AMD in the cross sectional analysis. The prevalence of AMD in this cohort was low compared to other cohorts in Europe, the US and Australia, and probably reflects the some selection biases in follow up participation as well as the low rate of smoking among our healthy participants.

## Introduction

Age-related macular degeneration (AMD) is a progressive chronic eye disease and the leading cause of low vision and blindness in developed countries.^1^ Early features include macular drusen—localised deposits of extracellular debris in Bruch’s membrane. The disease can progress to either geographic atrophy (“dry” AMD) or choroidal neovascularisation (“wet” AMD), both of which can have devastating effects on central vision in late stages of the disease. AMD causes approximately 5% of global blindness.[1] There are an estimated 71,000 new cases of late AMD per year in the UK[2], and projections suggest that 3 million Americans over the age of 50 will be affected by AMD by 2020[3]. Several genetic and environmental risk factors have been identified for AMD, with the strongest risk factor being older age. [4,5] Pooled findings from three continents showed that the prevalence of AMD was 0.2% in those aged 55–64 years compared to 13.1% in people aged 85 and over. [6]

Several studies have shown a consistent effect of cardiovascular risk factors on greater prevalence and incidence of AMD.[5,7–11] An adjusted analysis of results from the Beaver Dam, the Blue Mountains and Rotterdam Eye Studies showed that current smokers were more than twice as likely to develop late AMD than people who had never smoked. [4] Smoking is synergistic with genetic risk factors to increase risk of the disease.[12] Other identified vascular associations with AMD include cardiovascular disease,[10,13] stroke, [14] hypertension[10] and inflammatory markers such as CRP. [15,16] The evidence for the association between serum lipids and AMD is less consistent. The histological features of AMD suggest a possible role of lipids in the aetiology of AMD. Drusen are predominantly composed of lipids and proteins, and these deposits may interact with other lipids or proteins such as the complement complex in the disease progression pathway.[17] Advanced AMD is associated with variants in the hepatic lipase gene (LIPC) in the high-density lipoprotein (HDL) pathway.[18] However, the inconsistent direction of effects between other HDL-associated single-nucleotide polymorphisms (SNPs) and AMD suggests a complex relationship between HDL and AMD. Epidemiological studies have also provided mixed results, with studies showing no association[13], increased risk[19–22,23]and a protective association[24–26] of higher HDL cholesterol levels on AMD frequency.

In this study, we examined the cross sectional and longitudinal associations of cardiovascular risk factors and AMD.

## Methods

The European Prospective Investigation into Cancer (EPIC) and Nutrition study is a 10 country collaborative cohort study investigating lifestyle and nutritional risk factors for cancer. Detailed descriptions of the EPIC study methods and recruitment have been reported previously.[27,28,29] The baseline study cohort had lower smoking rates compared to national British population samples, [27] but were otherwise comparable with regard to anthropometry measures and blood pressure. The present study used exposure data collected from the first health examination (1993–1997) and the third health examination (2006–2011). The eye data were collected as part of the EPIC Norfolk Eye Study, which took place during the third round of clinical examinations and included a full ophthalmic examination.^36^ The study was approved by the Norfolk Local Research Ethics Committee and adhered to the Declaration of Helsinki. All participants gave written informed consent.

The health examinations were carried out by trained nurses using standard operating protocols. Systolic and diastolic blood pressure (sBP and dBP) were recorded as the mean of two measurements separated in time, taken from the right arm with the participant seated for 5 minutes, using an Accutorr Plus blood pressure monitor (Mindray, Huntingdon, UK). Height and weight were measured with participants dressed in light clothing and shoes removed. A stadiometer was used to measure height to the nearest 0.1metre (m), and the Tanita body composition analyser model TBF 300s (Chasmors Ltd, London) was used to measure weight to the nearest 100g. Body mass index (BMI) was calculated as weight in kg/height squared in m^2^. The measurement of habitual physical activity and visual acuity (VA) in EPIC-Norfolk has been previously described. The physical activity scale used has been validated against heart rate monitoring with individual calibration in independent studies.[30]

Serum high-sensitivity CRP was measured using the Olympus AU640 Chemistry Immuno Analyzer (Olympus Diagnostics, Watford, United Kingdom). Blood samples collected at baseline examination were centrifuged at 2,100*g* for 15 minutes at 4°C, and serum samples were kept frozen at −80°C until being thawed in 2008 for CRP assaying. Non-fasting serum total and high-density lipoprotein- cholesterol (HDL-C) levels (mmol/l) were measured at both health examinations using an RA 1000 Technicon analyser (Bayer Diagnostics, Basingstoke, UK). Low-density lipoprotein-cholesterol (LDL-C) was determined using the Friedewald formula. Social class was recorded according to the UK Registrar-General’s occupation-based classification system; this was based on the participant’s last occupation if they were retired. Educational level was recorded and classified into four groups according to the highest qualification achieved. The four categories are less than O levels, O levels, A levels and degree or above; O levels are qualifications gained at the end of secondary school after 11 years of schooling, and A levels after 13 years of schooling, with degrees obtained in university settings. Smoking status was recorded as current, former and never. The EPIC Norfolk database was linked to national hospital discharge data for Norfolk residents (ENCORE). Prevalent ischaemic heart disease (IHD) and stroke in this study were ascertained through self-rated disease at baseline (between 1993–1997), subsequent health examinations and any recorded episodes of IHD or cardiovascular accident (CVA) from hospital episodes statistics (HES) data accumulated from baseline until 2009.

### Ascertainment of AMD

Digital fundus photographs of the optic disc and macula were taken using a TRC-NW6S non-mydriatic retinal camera and IMAGEnet Telemedicine System (Topcon Corporation, Tokyo, Japan) with a 10 megapixel Nikon D80 camera (Nikon Corporation, Tokyo, Japan) without pharmacological dilation of the pupil. AMD was categorised by independent graders using a modified Wisconsin protocol[31]. Main features for each image were assessed with standardised photographs and included:

- Hard drusen size <63 μm, with more than ten hard drusen required to be present for the lesion to be classified as present
- Soft drusen of size ≥125μm, with presence of one soft drusen sufficient for classification of lesion to be present
- Geographic atrophy
- Choridal neovascularisation
- Retinal Pigment Epithelium (RPE) detachment
- Disciform scar

The predominant phenotype observed or the most severe lesion for each eye was used as the final grading for that eye. Individual categorisation of AMD lesion was based on the more severely affected eye.

### Statistical Analysis

The data were initially explored through descriptive analysis of variables using t-tests for quantitative and χ^2^ test for categorical variables to compare different groups. AMD was analysed as a binary variable with any AMD (including early disease) as the main outcome due to small numbers of people with advanced AMD. Smokers were dichotomised into “never” and “ever” for current and past smokers, due to the relatively small proportion of current smokers. A right skew was evident in the CRP distribution. Therefore, CRP was categorised into clinically relevant categories, ≤1, 1.1–3, 3.1–10, ≥10mg/L for initial descriptive and univariable analysis; subsequently both categorical CRP and a log transformed continuous CRP were analysed separately to maximise power to detect any differences. Univariable associations with any AMD as the dependent variable were explored using logistic regression, tabulation and χ^2^ test. A stepwise multivariable logistic regression model was used to examine the effect of detected and *a priori* risk factors on AMD. Odds ratio for AMD were estimated per 1 standard deviation (SD) increase in log transformed CRP. Indicator variables were used with all categorical variables in the multivariable analysis, with interaction terms to test for evidence of effect modification. All statistical analyses were conducted using STATA 12 (Statacorp, College Station, Texas, US).

## Results

Of 8,623 participants examined in the EPIC Norfolk Eye study, retinal photographs were obtained from 7,501 (87.0%) participants, of which 5,344 pairs of left and right eyes (62.0%) were of sufficient quality for grading of AMD related fundus lesions. Participants with missing or excluded photographs were older (70.8 years vs. 67.4 years p<0.01) and more likely to be male (47.5% of those missing vs 43.1% with gradable photographs p<0.01), with lower levels of education (28.4% of missing with no formal qualifications vs. 25.1% with graded photographs, p<0.01). Similar proportions of smokers did not have gradable photos compared to those with grading (8.6% vs 9.4% missing p = 0.23). The mean age of the 5,344 included participants was 67.4 years (range 48.4–91.9 years), with 56.9% women. Women were younger than men, with a mean age of 66.9 years compared to 68.1 years for men (p<0.01). Older men and women both had higher BMI, sBP, dBP, LDL-C, total cholesterol, triglycerides, and CRP. Older people were also more likely to have lower attained levels of education, to be less active, and more likely to have never smoked. Older men and women also had lower levels of HDL-C, though this was not statistically significant.

The study prevalence of AMD is shown in Table 1. There were 28 cases of late AMD (0.5%, 95% confidence interval (CI) = 0.3–0.8%) and 645 cases of early AMD (12.1%, 95%CI = 11.2–13.0.%). The vision in the worse eye of the 28 participants categorised with late AMD was 6/60 or worse in 3 cases (10.7%), 6/18-6/60 in 4 participants (14.3%) and the remaining 21 people (75.0%) had vision of 6/12 or better.

**Table 1:** AMD diagnoses in worse eye.

Type	Description	N	%
**No AMD**	No AMD or hard drusen only	4671	87.4
**Early AMD**	Soft drusen and/or pigmentary changes	645	12.1
**Late AMD**	Presence of either geographic atrophy or exudative macular degeneration	28	0.5
**Total**		5344	100.0

AMD = age related macular degeneration

Univariable associations between both baseline and follow up clinical variables and AMD diagnosed at the third health check are shown in Table 2. People with AMD were older and more likely to be female; they were also more likely to have higher levels of sBP, HDL-C and CRP measured at baseline. Similar associations were observed with follow up measurements. There were additional cross sectional associations with AMD and higher levels of triglycerides and physical activity.

**Table 2:** Univariable associations between cardiovascular risk factors measured at baseline and follow up and AMD in 5344 men and women.

	Baseline (1993–1997)			Follow up (2006–2011)		
	AMD	No AMD	p-value*	AMD	No AMD	p-value
Age (years)	57.7 (7.7)	54. (7.2)	<0.01	71.10 (7.7)	66.91 (7.5)	<0.01
BMI (kg/m2)	25.89 (3.64)	25.75 (3.7)	0.38	26.88 (4.10)	26.81 (4.36)	0.66
WHR	0.83 (0.09)	0.84 (0.09)	0.37	0.89 (0.08)	0.89 (0.08)	0.82
Weight	71.86 (12.1)	72.87 (12.8)	0.06	73.14 (13.4)	74.66 (14.2)	<0.01
Systolic Blood pressure (mmHg)	132.13 (16.9)	130.60 (16.4)	0.03	137.66 (17.3)	135.46 (16.6)	<0.01
Diastolic Blood pressure (mmHg)	80.85 (10.5)	80.93 (10.8)	0.85	78.31 (9.07)	78.58 (9.30)	0.49
HDL-cholesterol (mmol/L)	1.49 (0.42)	1.44 (0.42)	<0.01	1.58 (0.43)	1.50 (0.41)	<0.01
LDL-cholesterol (mmol/L)	3.84 (1.03)	3.82 (0.98)	0.53	3.14 (0.99)	3.23 (1.00)	0.04
Cholesterol (mmol/L)	6.06 (1.13)	5.99 (1.08)	0.19	5.38 (1.12)	5.45 (1.14)	0.18
Triglycerides (mmol/L)	1.62 (0.94)	1.67 (1.02)	0.28	1.70 (0.93)	1.69 (0.92)	<0.01
CRP (mg/L)			0.02			0.02
≤1.0	191 (41.0)	1617 (48.7)		103 (17.8)	721 (18.0)	
1.1–3.0	174 (37.3)	1100 (33.1)		284 (49.0)	2199 (54.8)	
3.0–10.0	85 (18.2)	520 (15.7)		165 (28.4)	917 (22.8)	
≥10	16 (3.4)	83 (2.5)		28 (4.8)	175 (4.4)	
Physical Activity			0.80			<0.01
Inactive	153 (22.7)	984 (21.1)		273 (41.2)	1584 (34.4)	
Moderately inactive	198 (29.4)	1419 (30.4)		189 (28.5)	1401 (30.4)	
Moderately active	175 (26.0)	1235 (26.4)		112 (16.9)	849 (18.4)	
Active	147 (21.8)	1033 (22.1)		89 (13.4)	777 (16.8)	
Smoking			0.17			0.32
Current	45 (6.7)	413 (8.9)		21 (3.2)	204 (4.4)	
Former	262 (39.1)	1751 (37.6)		298 (45.0)	2058 (44.6)	
Never	363 (54.2)	2488 (53.5)		344 (51.9)	2349 (51.0)	
Sex			0.009			
Male	259 (38.5)	2046 (43.8)				
Female	414 (61.5)	2625 (56.2)				
Social Class			0.94			
Non-manual	439 (65.8)	3050 (66.0)				
Manual	228 (34.2)	1573 (34.0)				
Education			0.08			
Less than O level	196 (29.1)	1143 (24.5)				
O Level	80 (11.9)	580 (12.4)				
A Level	282 (41.9)	2111 (45.2)				
Degree	115 (17.1)	836 (17.9)				
Total						

AMD = age-related macular degeneration, BMI = Body mass Index, HDL = high density lipoprotein, LDL = low density lipoprotein, CRP = C = reactive protein

*p-value from t-test or χ^2^ test for association between risk factor and AMD

Data presented as n(%) for categorical variables, and mean (SD) for continuous variables.

Although the results indicated that people with AMD were more likely to be inactive, the association was no longer statistically significant after adjusting for age and sex. At baseline, 50 people reported strokes, with 152 recorded by third health examination, with no evidence o f an association with AMD at any time point (0.5% of no AMD with stroke vs 0.9% AMD with stroke, p = 0.2 at baseline; 1.4% vs 1.6%, p = 0.6 in follow up). Similarly, 62 people had reported myocardial infarction at baseline with 161 at follow up with no associations observed with AMD (1.2% of no AMD with MI vs 0.9% AMD with MI, p = 0.5) at baseline or at the third health examination (3.0% no AMD with MI vs 3.4% with AMD had MI, p = 0.5).

In multivariable analysis shown in Table 3, older age was strongly associated with AMD, with 8% increase in odds of AMD per year older, adjusting for sex, education, sBP, HDL, smoking, and CRP. Higher sBP and female gender were no longer associated with AMD after adjusting for covariables. Although higher serum triglycerides were associated with AMD at follow up, it was not statistically significant in the final model, and removed due to its low impact on final estimates. Education and smoking were included in the final model as recognised markers of AMD risk, though no evidence of an association with AMD observed in this study sample. Both continuous CRP (using natural logs, OR presented in table is the exponential of the estimated coefficient) and HDL remained significantly associated with AMD in the final model using baseline measurements, with 1 SD increase in log transformed CRP (equating to approximately 3 fold increase in CRP) increasing odds of AMD by 11%, and 0.5 mmol/L increase in HDL increasing odds of AMD by 15%. In the final cross sectional model, CRP was not significantly associated with AMD, with only age and HDL remaining as independent risk factors. There was no evidence of an interaction with the categorical variables age (<60 vs >60), sex, CRP categories, smoking, social class or education when interaction terms were used (all interaction tests p>0.05).

**Table 3:** Multivariable logistic regression results for odds of AMD detected in Third health examination for variables collected at baseline and follow up.

	Baseline				Follow up			
	OR*	95% CI		p-value^‡^	OR*	95% CI		p-value^‡^
Age per year	1.08	1.06	1.09	<0.01	1.07	1.06	1.09	<0.01
Female	1.21	0.96	1.54	0.11	1.18	0.93	1.50	0.18
**Education Level**				0.92				0.84
Less than O level	Ref				Ref			
O level	1.00	0.70	1.42	1.00	0.94	0.66	1.34	0.73
A level	0.95	0.74	1.22	0.68	0.89	0.69	1.15	0.36
Degree	1.05	0.76	1.43	0.79	0.94	0.68	1.30	0.71
Systolic blood pressure per 20mmHg	0.94	0.83	1.06	0.31	1.03	0.92	1.17	0.59
HDL cholesterol per 0.5mmol/L	1.15	1.01	1.30	0.03	1.26	1.10	1.43	<0.01
Smoking				0.95				0.98
Current	Ref				Ref			
Former	1.02	0.67	1.53	0.94	0.95	0.54	1.68	0.86
Never	0.98	0.66	1.47	0.93	0.95	0.54	1.67	0.85
CRP^†^(mg/L)	1.11	1.00	1.23	0.04	1.10	1.00	1.22	0.08

OR = Odds ratio, CI = confidence interval, HDL = High density lipoprotein, CRP = C reactive protein

*All variables were mutually adjusted in one regression model;

^‡^p-value from Wald test or likelihood ratio test for categorical variables.

^†^Per 1 SD increase in log transformed CRP (equating to approximately 3 fold increase in CRP)

## Discussion

In this community based study of 5344 older people, we found that higher levels of HDL and CRP at baseline, but neither smoking nor other cardiovascular risk factors, were associated with a higher occurrence of AMD. HDL was the only cardiovascular risk factor associated with AMD in the cross sectional analysis.

There was an overall low study prevalence of AMD, with only 12.6% (95%CI = 11.7–13.5%) of participants with signs of AMD and 0.5% (95%CI = 0.3–0.7%) with geographic atrophy or NV-AMD. This was lower than the prevalence reported in epidemiological studies in predominantly White populations of the United States (US), Australia and the Netherlands, where late AMD (geographic atrophy or neovascular AMD) was observed in 1.6, 1.9 and 1.7% respectively.[32–34] The lower prevalence in our study was likely due to a selection bias from healthy survivors of a longitudinal study who were able to attend a clinic examination; the implications of this will be discussed further.

The observed association with baseline CRP was consistent with findings from a review of observational studies by Hong et al.,[16] where the pooled OR from the meta-analysis of studies examining low vs high levels of CRP (commonly <1.1 vs ≥3mg/L) using both early and late AMD as an outcome was 1.31 (95% CI = 1.04–1.65), which is similar to our results (OR = 1.11 95%CI = 1.00–1.23 for a nearly 3 fold increase). There is substantial evidence to support an inflammatory mechanism in the pathogenesis of AMD. A variety of raised inflammatory markers and genes in the complement pathway, including complement factor H (*CFH*),[35,36] complement factor B/complement component 2 (*CFB/C2*),[37] complement 3 (*C3*),[38] and complement factor I (*CFI*)[39] are associated with increased risk of AMD. In the cross sectional examination, CRP was no longer associated with AMD after adjusting for age and sex. These differing associations are in line with findings from Hong’s meta-analysis stratified by study design, where pooled analysis of population based longitudinal studies showed a significant association with AMD (pooled OR = 2.20, 95%CI = 1.24–3.91) though the population based cross sectional studies did not (pooled OR = 1.22 (0.93–1.60). The authors suggested that underlying differences in ascertainment of AMD were the main reason for the differences; our results indicate otherwise. One possible explanation is that different levels of CRP mark different stages of the disease process, with inflammatory processes contributing to the initial onset or stages of disease and lower levels of inflammation in established disease.

Previous reports on the association between HDL and AMD have been inconsistent, with studies showing positive, inverse or no association.[13,19,24] We found that higher levels of HDL at baseline (and likely lower cardiovascular risk) were associated with higher odds of AMD (OR = 1.37, 95% CI = 1.05–1.78), but there was no association between AMD and LDL; this supports findings from the POLA study that found a 50% increased risk with higher levels of HDL.[20] Pooled data from the three epidemiological studies with predominantly White populations did not find a significant association with HDL (OR = 1.00, 95%CI = 0.98–1.03).[4] These conflicting findings are reflected in the complex associations with HDL pathway genes, where alleles of the LIPC gene that raise HDL and alleles of the CEPT genes that decrease HDL have both been implicated in AMD aetiology.[18] It is possible that observed differences might be due to differences in the background environmental and genetic risk in the study populations. HDL has heterogeneous structure and functions, with normal functions including cholesterol lowering and anti-inflammatory properties. However, inflammation can modify the structure of HDL and lowers its ability to reduce peripheral cholesterol and it is transformed to a pro-inflammatory particle. [40] Recent studies have shown that high levels of HDL are associated with an increased risk of recurrent coronary events in patients with previous infarcts.[41] Furthermore, follow up of this cohort showed that those with high levels of HDL and CRP at baseline were at increased risk of incident CVD. [42] The interaction between baseline CRP and HDL could explain our results. However, there was no evidence of a statistical interaction between baseline HDL and dichotomised CRP (<3 vs > = 3mmol/l) levels for risk of AMD, though this does not preclude a potential effect due to the low power of the interaction test.

We did not detect an association between smoking and AMD, which contrasts strongly with several epidemiological studies and meta- analyses where smoking is associated with a two fold increase in neovascular AMD risk.[4] Epidemiological studies have shown stronger effects with current smokers compared to past smokers.[7,10] Pooled analysis from the Beaver Dam, Blue Mountains and Rotterdam eye studies showed a longitudinal association between current smokers at baseline and the 5–6 year incidence and progression of early AMD.[4] Overall, 18.5% of participants with follow up in the 3 studies were smokers at baseline compared to 8.6% in our selected study population. Assuming an OR of 2.3, the current study proportions of late AMD and current smokers would have only had 16% power to detect an effect.

There were limitations in our study. The present study was an average 17 year follow up of a larger longitudinal study, with healthier survivors and a lower frequency of risk factors and outcomes, which would reduce the likelihood of detecting true associations. People with AMD or cardiovascular disease were also less likely to attend due to relatively poor health and/or vision. However, both factors resulting in truncation of the distribution would be likely to attenuate any underlying associations. In addition to reduced power, there may also have been measurement error in assessment of AMD. However, the use of a standardised objective grading system would have mitigated potential systematic errors. We also used non-mydriatic photographs, and 28.8% of photos were of insufficient quality for grading. Those with ungradable photos were older and more likely to have AMD, further reducing power. The predominant phenotype approach may have reduced detection of small differences between clinical subtypes, but would have increased reliability of grading outcomes. We were only able to ascertain the outcome in the follow up study and cannot exclude prevalent cases at baseline, however, as there was a very low prevalence of late AMD In the follow up, there were unlikely to have been many cases of AMD at baseline. Furthermore, excluding late cases does not alter the interpretation of the final model, indicating that baseline risk factors were likely to have been present prior to disease, and also the associations detected relate primarily to early AMD. Additional cardiovascular risk factors known to be associated with AMD such as dietary intake of oily fish and intake of leafy green vegetables were not included in this analysis because it was beyond the scope of the current investigation, which focused on more proximal markers of cardiovascular risk. Further research into dietary factors and AMD are important because they are modifiable risk factors and could inform public health action in the prevention of AMD.

In summary, we have found that older age, higher baseline and follow up levels of HDL, and baseline CRP were associated with increased odds of AMD. The prevalence of AMD in this cohort was low compared to other cohorts in Europe, the US and Australia, and possibly reflects healthier participants as well as the low rate of smoking among our participants.
